# Supplementary material for: Unraveling the causal genes and transcriptomic determinants of human telomere length
Source: Nat Commun. 2023 Dec 21;14:8517. doi: 10.1038/s41467-023-44355-z (PMC10739845; doi:10.1038/s41467-023-44355-z)
Supplement: Supplementary file 1 — Supplementary Information [file 41467_2023_44355_MOESM1_ESM.pdf]

Supplementary Materials

Unraveling the causal genes and transcriptomic determinants of human telomere length

This file includes:
Supplementary Figures S1 to S14

Supplementary Figures

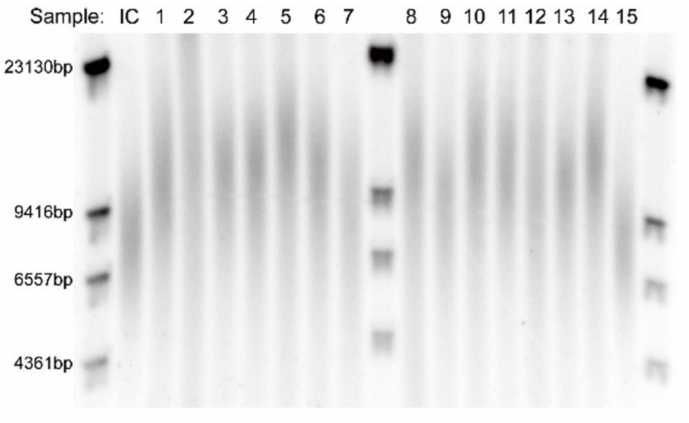

Supplementary Figure 1. Terminal restriction fragment (TRF) measurement of telomere length (TL) on placental tissues. TL of 15 randomly selected placental tissue samples was tested using the TRF-based method with pulsed-field electrophoresis.

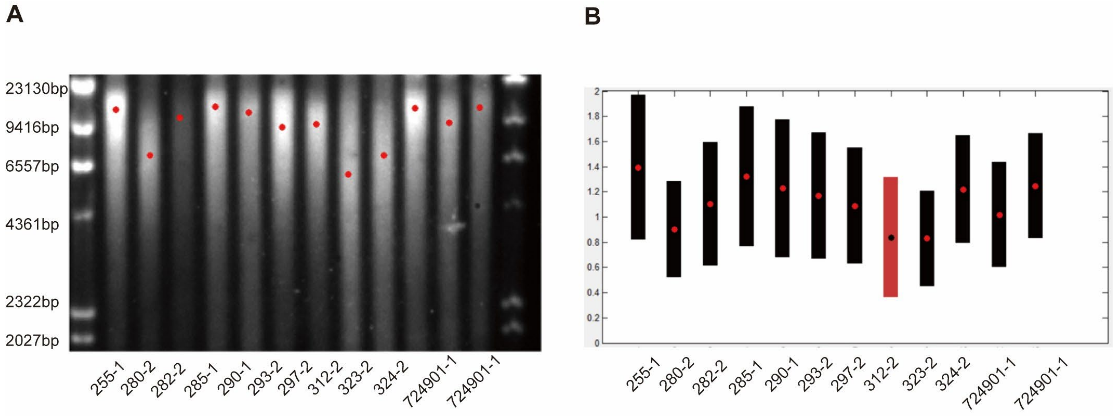

Supplementary Figure 2. Estimate of variation within the TRF measurements based on repeated samples. A. Examples of southern blot was conducted to analysis the telomere length of samples obtained from the placenta; B. Telomere length analysis was performed using TeloTool software with a threshold set at 60%. The black bars represent the analyses that met the fitting criteria, while the red bars represent the analyses that did not meet the fitting criteria and will be excluded from the subsequent statistical analysis.

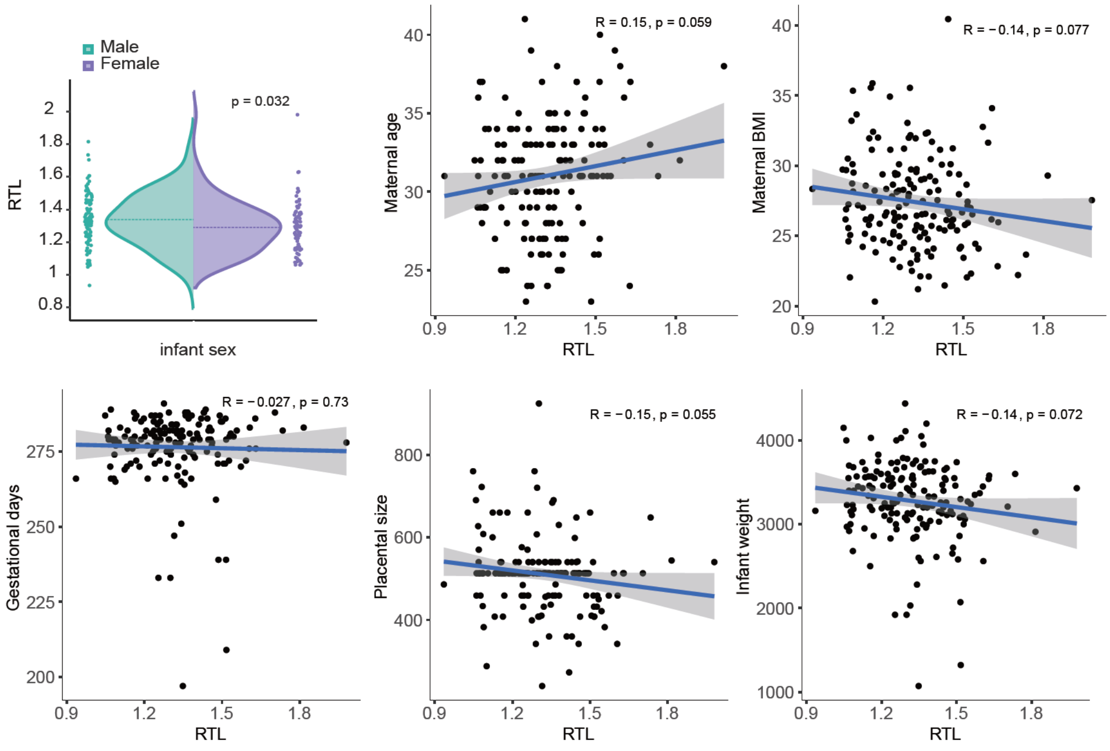

Supplementary Figure 3. Pearson correlation between demographic factors and relative telomere length (RTL). The scatter plot showing the correlations between RTL and demographic factors (including infant sex, maternal age, maternal BMI, gestational days, placental size, and infant weight), with a simple linear regression line fitted. For the violin plot, male (n=63), female (n=73). For the scatter plot, *P* values were computed by the two-sided *P*-value of Pearson's correlation test, no adjustments for multiple comparisons.

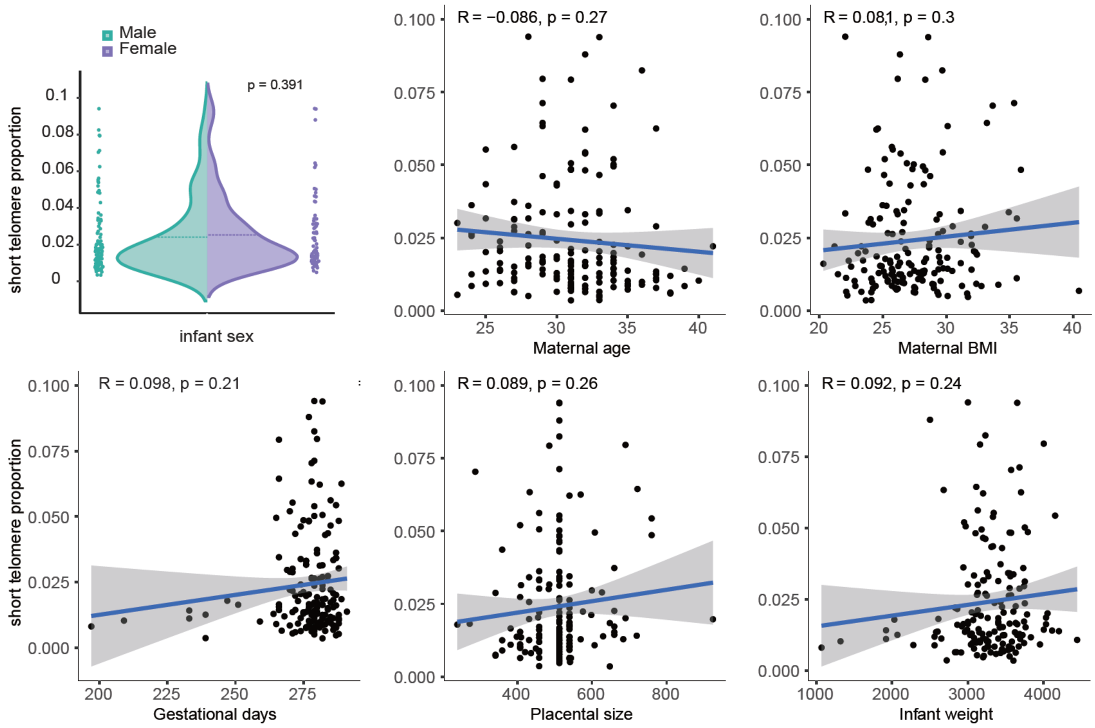

Supplementary Figure 4. Pearson correlation between demographic factors and short telomere proportion (STP). The scatter plot showing the correlations between STP and demographic factors (including infant sex, maternal age, maternal BMI, gestational days, placental size, and infant weight), with a simple linear regression line fitted. For the violin plot, male (n=63), female (n=73). For the scatter plot, *P* values were computed by the two-sided *P*-value of Pearson's correlation test, no adjustments for multiple comparisons.

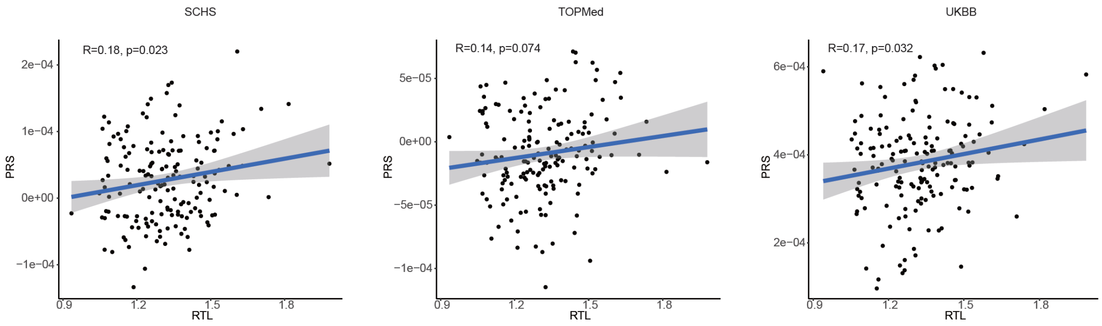

Supplementary Figure 5. Pearson correlation between polygenic risk scores (PRS) and RTL based GWAS results from different cohorts. The scatter plot showing the correlations between RTL and PRS based on TL GWAS summary statistics of single cohort (SCHS, TOPMed, and UKBB), with a simple linear regression line fitted. *P* values were computed by the two-sided *P*-value of Pearson's correlation test, no adjustments for multiple comparisons.

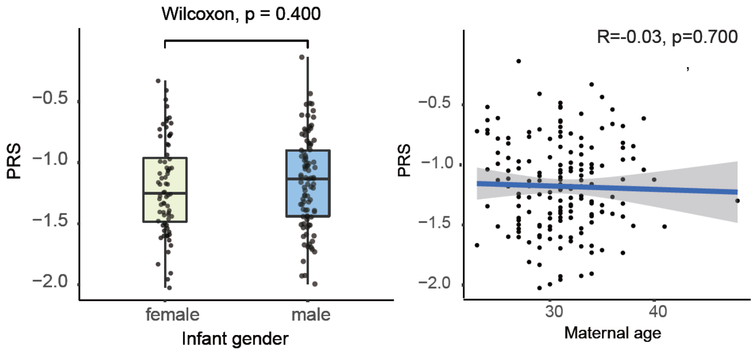

Supplementary Figure 6. Comparison of PRS with infant gender/maternal age. The box plot compares the PRS in female infants versus male infants, and the scatter plot showing the correlations between maternal age and PRS, with a simple linear regression line fitted. For the box plot, *P* values were computed by the Wilcoxon test. For the scatter plot, *P* values were computed by the two-sided *P*-value of Pearson's correlation test, no adjustments for multiple comparisons.

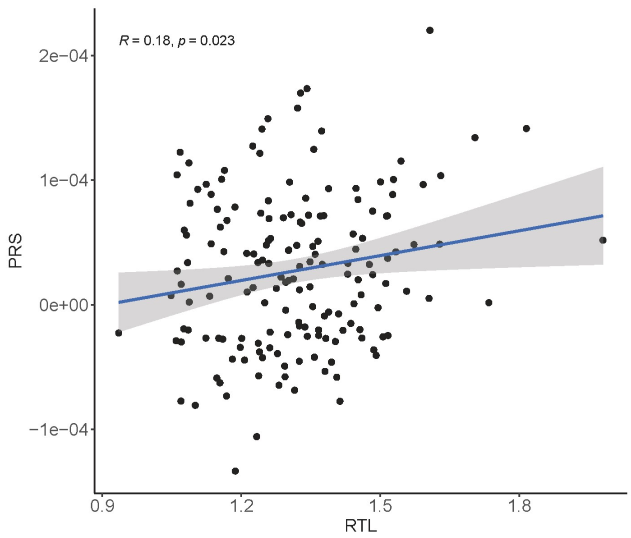

Supplementary Figure 7. TL GWAS and PRS analysis. Scatter plot showing the correlations between PRSs based on GWAS hits from the trans-ethnic analysis that exhibited nominal (*P*-value < 0.05) associations in the SCHS study and RTL, with a simple linear regression line fitted. *P* value was computed by the two-sided *P*-value of Pearson's correlation test.

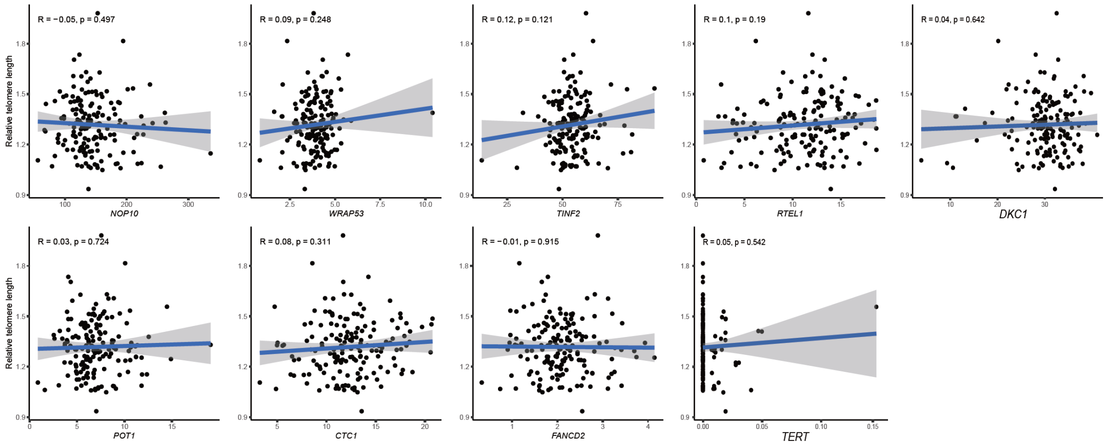

Supplementary Figure 8. Pearson correlation between genes encoding telomerase components or genes encoding telomere-binding proteins and RTL. The scatter plot showing the correlations between genes encoding telomerase components or genes encoding telomere-binding proteins and RTL, with a simple linear regression line fitted. *P* values were computed by the two-sided *P*-value of Pearson's correlation test, no adjustments for multiple comparisons.

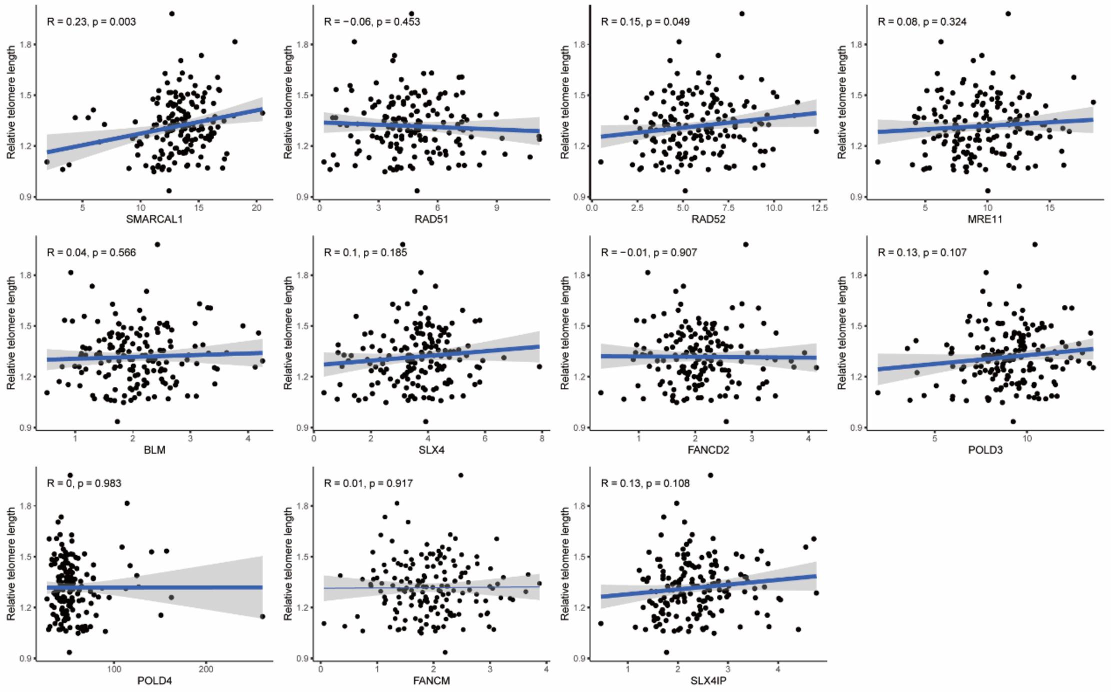

Supplementary Figure 9. Pearson correlation between genes involved in Alternative Lengthening of Telomeres (ALT) pathways and RTL. The scatter plot showing the correlations between genes involved in ALT pathways (expect for *ATRX* and *DAXX*, showed in Figure 3) and RTL, with a simple linear regression line fitted. *P* values were computed by the two-sided *P*-value of Pearson's correlation test, no adjustments for multiple comparisons.

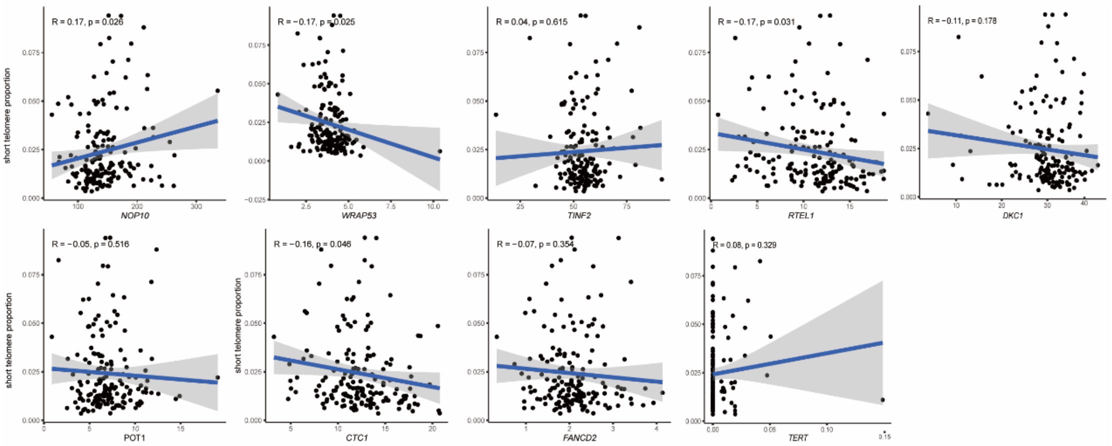

Supplementary Figure 10. Pearson correlation between genes encoding telomerase components or genes encoding telomere-binding proteins and STP. The scatter plot showing the correlations between genes encoding telomerase components or genes encoding telomere-binding proteins and STP, with a simple linear regression line fitted. *P* values were computed by the two-sided *P*-value of Pearson's correlation test, no adjustments for multiple comparisons.

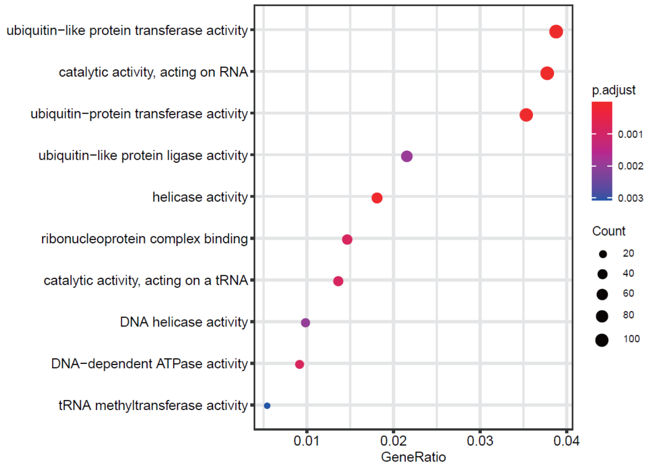

Supplementary Figure 11. Functional enrichment for genes associated with a WGCNA co-expression module. Dot plot of GO enrichment for genes clusters in turquoise module. The diameter indicates the number of genes overlapping the gene ontology term and the color indicates the BH adjusted enrichment *P*-value.

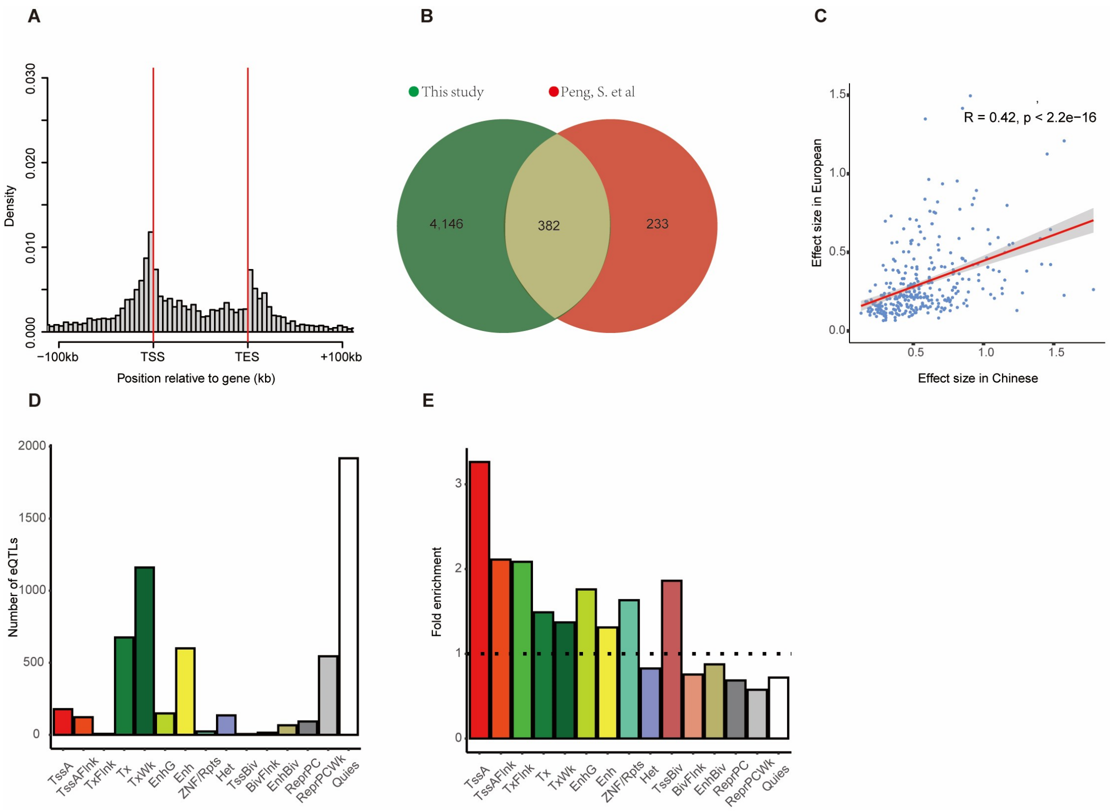

Supplementary Figure 12. Characteristics of placental eQTLs. (A) The distributions of genomic distances from eQTLs to the gene transcription start site/end site to target genes. (B) Venn plot of eGenes detected in this study and in Peng, S. *et al.* 2017 *Hum Mol Genet.* (C) The effect sizes of eQTLs towards target genes in the Chinese cohort (X axis) and European cohort (Y axis) are also correlated. *P* value was computed by the two-sided *P*-value of Pearson's correlation test. (D) Barplots shows that the most of the placental eQTLs are located in chromatin active regions. (E) Barplots shows that placental eQTLs are especially enriched in placental active promoter (TssA), and enhancer (Enh and EnhG) regions.

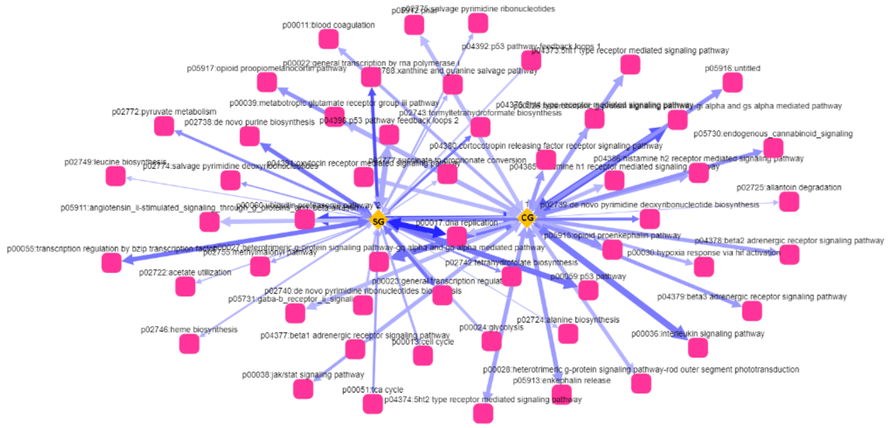

Supplementary Figure 13. Network enrichment against signature genes and TL-causal genes using EviNet algorithm. Yellow diamonds: gene sets. Red boxes: pathways. Two-headed arrows: summaries of individual gene-gene links from the global network that connected signature genes and causal genes. Edge thickness: the number of gene-gene links.

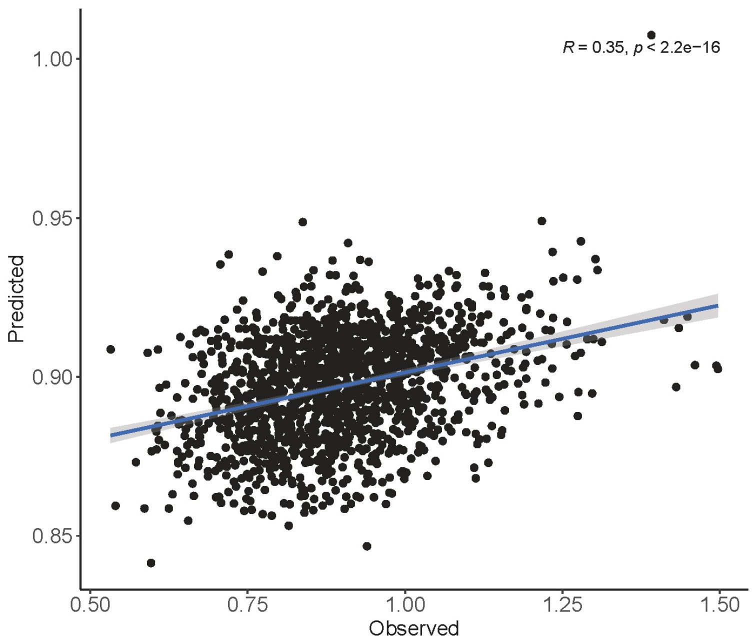

Supplementary Figure 14. Pearson correlation between the actual and predicted TL values. Scatter plot shows the actual TL values against the values predicted by the model based on UKBB Chinese data. *P* value was computed by the two-sided *P*-value of Pearson's correlation test.
