# Supplementary material for: Unraveling the causal genes and transcriptomic determinants of human telomere length
Source: Nat Commun. 2023 Dec 21;14:8517. doi: 10.1038/s41467-023-44355-z (PMC10739845; doi:10.1038/s41467-023-44355-z)
Supplement: Supplementary file 3 — Description of Additional Supplementary Files [file 41467_2023_44355_MOESM3_ESM.pdf]

Description of Additional Supplementary Files

File Name: Supplementary Data 1
Description: Demographic information of the participants.

File Name: Supplementary Data 2
Description: Incorporated TL-related GWASs for the trans-ancestral meta-analysis.

File Name: Supplementary Data 3
Description: New genetic loci identified in trans-ancestral TL GWAS.

File Name: Supplementary Data 4
Description: All significant TL-associated loci identified in trans-ancestral TL GWAS.

File Name: Supplementary Data 5
Description: All significant TL-associated loci reported by SCHS, TopMed, and UKBB.

File Name: Supplementary Data 6
Description: Variant-level associations for each of the significant trans-ancestral GWAS hits with placental RTL.

File Name: Supplementary Data 7
Description: The Pearson correlations between tested genes and RTL.

File Name: Supplementary Data 8
Description: The Pearson correlations between tested genes and STP.

File Name: Supplementary Data 9
Description: Colocalization results with different level of evidence.

File Name: Supplementary Data 10
Description: TWAS results for genes with FDR < 0.1.

File Name: Supplementary Data 11
Description: SMR results for genes with FDR < 0.1.

File Name: Supplementary Data 12
Description: The prioritized causal genes related to TL.

File Name: Supplementary Data 13
Description: Correlation between the predicted RTL and the observed RTL measured by Luminex-based assay across different GTEx tissues.
