# Supplementary material for: Unraveling the causal genes and transcriptomic determinants of human telomere length
Source: Nat Commun. 2023 Dec 21;14:8517. doi: 10.1038/s41467-023-44355-z (PMC10739845; doi:10.1038/s41467-023-44355-z)
Supplement: Supplementary file 5 — Reporting Summary [file 41467_2023_44355_MOESM5_ESM.pdf]

nature portfolio

Corresponding author(s): Mulin Jun Li
Last updated by author(s): Nov 19, 2023

Reporting Summary

Nature Portfolio wishes to improve the reproducibility of the work that we publish. This form provides structure for consistency and transparency in reporting. For further information on Nature Portfolio policies, see our Editorial Policies and the Editorial Policy Checklist.

Statistics

For all statistical analyses, confirm that the following items are present in the figure legend, table legend, main text, or Methods section.

n/a	Confirmed	
☐	☒	The exact sample size (*n*) for each experimental group/condition, given as a discrete number and unit of measurement
☐	☒	A statement on whether measurements were taken from distinct samples or whether the same sample was measured repeatedly
☐	☒	The statistical test(s) used AND whether they are one- or two-sided<br>*Only common tests should be described solely by name; describe more complex techniques in the Methods section.*
☐	☒	A description of all covariates tested
☐	☒	A description of any assumptions or corrections, such as tests of normality and adjustment for multiple comparisons
☐	☒	A full description of the statistical parameters including central tendency (e.g. means) or other basic estimates (e.g. regression coefficient) AND variation (e.g. standard deviation) or associated estimates of uncertainty (e.g. confidence intervals)
☐	☒	For null hypothesis testing, the test statistic (e.g. *F*, *t*, *r*) with confidence intervals, effect sizes, degrees of freedom and *P* value noted<br>*Give P values as exact values whenever suitable.*
☒	☐	For Bayesian analysis, information on the choice of priors and Markov chain Monte Carlo settings
☒	☐	For hierarchical and complex designs, identification of the appropriate level for tests and full reporting of outcomes
☐	☒	Estimates of effect sizes (e.g. Cohen's *d*, Pearson's *r*), indicating how they were calculated

Our web collection on statistics for biologists contains articles on many of the points above.

Software and code

Policy information about availability of computer code

Data collection	The data associated with the curated genome-wide studies which collected from PubMed and literature, are listed at Supplementary Data.
Data analysis	All software used for data analysis are publicly available.<br>R (v4.0.3) R Core Team, 2017 https://www.r-project.org/<br>FastQC (v0.11.9) Software http://www.bioinformatics.babraham.ac.uk/projects/fastqc<br>STAR (v2.5.3a) Software https://github.com/alexdobin/STAR<br>UKBB (v3) UK BIOBANK http://www.nealelab.is/uk-biobank<br>GENCODE V26 Frankish et al., 2019 https://www.gencodegenes.org/<br>RNA-SeqQC (v1.1.9) Software https://github.com/getzlab/rnaseqc<br>WGCNA (v1.70.3) https://cran.r-project.org/web/packages/WGCNA/index.html<br>clusterProfiler (v4.6.0) https://bioconductor.org/packages/release/bioc/html/clusterProfiler.html<br>tensorQTL Software https://github.com/broadinstitute/tensorqtl<br>EIGENSTRAT (v7.2.0) Software https://github.com/DReichLab/EIG<br>NIH Roadmap Epigenomics https://egg2.wustl.edu/roadmap/<br>METAL Software https://genome.sph.umich.edu/wiki/METAL_Documentation<br>CMplot (v3.7.0) Software https://github.com/YinLiLin/CMplot<br>1000 Genomes Project (Phase 3) https://www.internationalgenome.org/<br>PRSice-2 (v2.3.3) Software https://choishingwan.github.io/PRS-Tutorial/prsice/<br>COLOC (v3.2.1) Software http://coloc.cs.ucl.ac.uk/coloc/<br>locuszoom Software http://locuscompare.com/<br>FUSION Software http://gusevlab.org/projects/fusion

SMR (v1.0.3) Software https://yanglab.westlake.edu.cn/software/smr/
TelSeq (v0.0.1) Software https://github.com/zd1/telseq
GraphPad Prism (v8.1.1) GraphPad Software https://www.graphpad.com/

For manuscripts utilizing custom algorithms or software that are central to the research but not yet described in published literature, software must be made available to editors and reviewers. We strongly encourage code deposition in a community repository (e.g. GitHub). See the Nature Portfolio guidelines for submitting code & software for further information.

Data

Policy information about availability of data

All manuscripts must include a data availability statement. This statement should provide the following information, where applicable:

- Accession codes, unique identifiers, or web links for publicly available datasets
- A description of any restrictions on data availability
- For clinical datasets or third party data, please ensure that the statement adheres to our policy

The raw RNA-seq and genotyping data are protected and are not available due to data privacy laws. The processed RNA-seq data, full summary statistics of eQTL and GWAS meta-analysis generated in this study are available at Figshare (https://figshare.com/s/f6de1a56ad7c448c1f4c). The TRF-based TL measurement of placenta samples generated in this study are provided in the Source Data. The individual-level genotypes of UKBB samples are available by application to the UKBB (https://www.ukbiobank.ac.uk/register-apply/). The data associated with the curated genome-wide studies which collected from PubMed and literature, are listed at Supplementary Data. The full GWAS summary statistics for TOPMed are available in the database of Genotypes and Phenotypes (dbGaP), under accession code phs001974.v3.p1[https://www.ncbi.nlm.nih.gov/projects/gap/cgi-bin/study.cgi?study_id=phs001974.v3.p1]. The full GWAS summary statistics for SCHS are available at Figshare (https://doi.org/10.6084/m9.figshare.8066999). The full GWAS summary statistics for UKBB data used in this study are available in the https://figshare.com/s/caa99dc0f76d62990195. The TL data of various tissues in GTEx are available in the (https://www.gtexportal.org/home/datasets). The WGS data of GTEx Whole blood samples are available in the dbGaP, under accession code phs000424.v8.p2 [https://www.ncbi.nlm.nih.gov/projects/gap/cgi-bin/study.cgi?study_id=phs000424.v8.p2].

Research involving human participants, their data, or biological material

Policy information about studies with human participants or human data. See also policy information about sex, gender (identity/presentation), and sexual orientation and race, ethnicity and racism.

Reporting on sex and gender	Among the collected placenta samples, 73 were females and 93 were males. The blood samples comprised 143 males and 88 females. Skin samples were obtained from healthy individuals (6 males and 6 females). Among the 1450 UKBB Chinese samples, 907 were females and 543 were males.
Reporting on race, ethnicity, or other socially relevant groupings	*Please specify the socially constructed or socially relevant categorization variable(s) used in your manuscript and explain why they were used. Please note that such variables should not be used as proxies for other socially constructed/relevant variables (for example, race or ethnicity should not be used as a proxy for socioeconomic status).*<br>*Provide clear definitions of the relevant terms used, how they were provided (by the participants/respondents, the researchers, or third parties), and the method(s) used to classify people into the different categories (e.g. self-report, census or administrative data, social media data, etc.)*<br>*Please provide details about how you controlled for confounding variables in your analyses.*
Population characteristics	The average age of the healthy singleton Chinese pregnancies was 32 ± 4.0 years, and no tobacco-smoking or alcohol-drinking behavior was noted. The blood samples comprised 143 males and 88 females, with an average age of 46.9 years (ranging from 5 to 89 years).Skin samples were obtained from healthy individuals (6 males and 6 females) with an average age of 37.6 years (ranging from 26 to 59 years). Among the 1450 UKBB Chinese samples, 907 were females and 543 were males with an average age of 64 years (ranging from 50 to 83 years).
Recruitment	The healthy singleton Chinese pregnancies (n = 166) were recruited prior to delivery at Tianjin Central Hospital of Gynecology Obstetrics, China. These study participants did not have any recorded medical disorders or adverse pregnancy outcomes. All participants provided written informed consent before sample collection. Placentas were treated within 10min of a vaginal delivery from full-term pregnancies (37+0–41+6 weeks). The average age of the participants was 32 ±4.0 years, and no tobacco-smoking or alcohol-drinking behavior was noted. The blood samples comprised 143 males and 88 females, with an average age of 46.9 years (ranging from 5 to 89 years). Peripheral blood samples of 0.5-2.5ml were collected from the elbow vein of each participant and preserved in EDTA-coated venous blood collection tubes for subsequent genomic DNA extraction. Additionally, 12 skin samples were obtained from healthy individuals (6 males and 6 females) with an average age of 37.6 years (ranging from 26 to 59 years), and genomic DNA was extracted for further telomere length analysis. Furthermore, the study included normal adult cardiac and lung tissues (N=6 each) for telomere length analysis. Ethical regulations were strictly followed, and the use of fetal samples was approved under protocol number 2022ky071-1. Approximately 100mg tissue samples were used for genomic DNA extraction.
Ethics oversight	The Tianjin Central Hospital of Gynecology Obstetrics ethics committees approved the collection and use of human placenta, skin, blood, cardiac, and lung samples.

Note that full information on the approval of the study protocol must also be provided in the manuscript.

Field-specific reporting

Please select the one below that is the best fit for your research. If you are not sure, read the appropriate sections before making your selection.

☒ Life sciences ☐ Behavioural & social sciences ☐ Ecological, evolutionary & environmental sciences

For a reference copy of the document with all sections, see nature.com/documents/nr-reporting-summary-flat.pdf

Life sciences study design

All studies must disclose on these points even when the disclosure is negative.

Sample size	Sample size were both indicated in the legend captions manuscript or the parametric results for all individuals for each sample plotted.
Data exclusions	No data were excluded from analyses.
Replication	Experiments described in the manuscript were performed at least three times as indicated in the figure legends unless otherwise mentioned. All attempts at replication were successful and all experimental findings were replicably reproduced.
Randomization	There was no bias in the selection of samples.
Blinding	Investigators were blinded to experimental groups as data collection, and quantification was objective and not impacted by investigators presumptions.

Reporting for specific materials, systems and methods

We require information from authors about some types of materials, experimental systems and methods used in many studies. Here, indicate whether each material, system or method listed is relevant to your study. If you are not sure if a list item applies to your research, read the appropriate section before selecting a response.

Materials & experimental systems

n/a	Involved in the study	
☒	☐	Antibodies
☐	☒	Eukaryotic cell lines
☒	☐	Palaeontology and archaeology
☒	☐	Animals and other organisms
☒	☐	Clinical data
☒	☐	Dual use research of concern
☒	☐	Plants

Methods

n/a	Involved in the study	
☒	☐	ChIP-seq
☒	☐	Flow cytometry
☒	☐	MRI-based neuroimaging

Eukaryotic cell lines

Policy information about cell lines and Sex and Gender in Research

Cell line source(s)	HTR-8/Svneo (cat# CRL-3271) and 293T (cat# CRL-3216) cells were purchased from the American Type Culture Collection (ATCC) (Virginia, USA).
Authentication	Short tandem repeat authentication services from ATCC were used to authenticate the cell lines.
Mycoplasma contamination	The cell lines were regularly tested for mycoplasma and were found to be negative.
Commonly misidentified lines (See ICLAC register)	No commonly misidentified cell lines were used in this study.
